# Zinc, oxidative stress, genetic background and immunosenescence: implications for healthy ageing

**DOI:** 10.1186/1742-4933-3-6

**Published:** 2006-06-26

**Authors:** Eugenio Mocchegiani, Marco Malavolta, Fiorella Marcellini, Graham Pawelec

**Affiliations:** 1 Immunology Ctr. (Section: Nutrition and Immunosenescence) Res. Dept. INRCA, Ancona, Italy; 2 Social Gerontology Unit, Res. Dept. INRCA, Ancona, Italy; 3 Center for Medical Research, University of Tuebingen, D-72072 Tuebingen, Germany

## Abstract

The relevance of zinc for proper functioning of the entire immune system is already well documented. However, the identification of individuals who really need zinc supplementation is still debated in view of the fact that excessive zinc may also be toxic. The risk of developing zinc deficiency in people from industrialized countries is relatively low, except for elderly subjects where zinc intake may be suboptimal and inflammation is chronic. Thus, the role of zinc on the immune system and on the health of European elderly people is becoming of paramount importance, considering also that the elderly population is rapidly increasing. In particular, the factors contributing to and the biochemical markers of zinc deficiency in the elderly are still remain to be established. Epidemiological, functional, and genetic studies aimed at formulating a rationale for the promotion of healthy ageing through zinc supplementation was the subject of an International Conference held in Madrid from 11–13 February 2006 (3^rd ^ZincAge Meeting) at the **CNIO Institute **(local organizer: **Maria Blasco, **partner of ZincAge)

## Introduction

**ZincAge **[1] is a specific targeted research project (STREP) funded by the European Union in the 6th Framework Program (FP6). It involves epidemiological studies on the influence of diet and lifestyle on healthy ageing, aimed at preventing adult degenerative disease, particularly focusing on cardiovascular diseases and also addressing malnutrition of the elderly. The 3^rd ^ZincAge meeting brought together biochemical, genetic and lifestyle implications for healthy ageing in the context of nutritional impact of zinc.

It is quite clear that antioxidant and micronutrients in the diet, such as zinc, influence the development and function of immune cells, the activity of stress-related proteins and antioxidant enzymes and help to maintain genomic integrity and stability. All these functions occur through the action of proteins involved in the regulation of zinc homeostasis, such as metallothioneins (MT), which bind zinc with high affinity but, at the same time, release free zinc ions in response to oxidative/nitrosative stress to modulate the expression of zinc-dependent genes and to activate antioxidant enzymes and impact immune response. Thus, the role of zinc is mainly to transduce oxidative stress and other signals converging at the production of nitric oxide into an specific intracellular response, suggesting an intriguing task of "signal transducer", similar to that found for calcium in the past. However, many aspects of this model are still unexplored, because the intracellular mechanisms involved in the regulation zinc homeostasis have been poorly studied in ageing.

It is known is that during ageing, the intake of zinc decreases, thus contributing to cause frailty, general disability and increased incidence of age-related degenerative diseases (cancer, infections and atherosclerosis). This situation may be better or worse in different European countries, taking into account the large differences in dietary habits between southern and northern areas. One of the aim of the ZincAge project is therefore to investigate how different dietary habits and intrinsic factors (genetic background) contribute to the risk of developing zinc deficiency in different European countries [1]. Considering all these aspects, the meeting was divided into 5 sessions.

### Session 1) Zinc, genetic background and brain

Chronic non-specific inflammation and immunopathology, which are thought to be a major problem in ageing, contribute significantly to the decline of the zinc status possibly aggravated by an unfavourable genetic background concerning genes involved in the inflammatory response (such as TNF-alpha and IL-6) and in the maintenance of zinc homeostasis, such as metallothioneins (MT). This means that in order to perform an appropriate supplementation with zinc in the elderly, it is necessary to consider zinc status, dietary habits and the individual genetic background. This last point may be crucial also in order to identify subjects that are at higher risk of developing zinc deficiency in ageing. In such subjects, there is the potential for a preventive intervention with zinc supplementation as suggested by **Eugenio Mocchegiani (INRCA, Ancona, Italy) (Coordinator of the project) **[2] who reported on the existence of differences in zinc status, measured by total plasma zinc, available intracellular and plasma zinc and MT, in elderly individuals recruited in different European countries (Italy, France, Germany, Poland and Greece). This was found to be related to different dietary habits and to the presence of particular polymorphisms of IL-6 and MT genes, thus confirming that the rationale for zinc supplementation should take these factors into account. This issue was further extended in the "young scientists session" of the Meeting by **Catia Cipriano (INRCA, Ancona, Italy), **who showed the relevance of MT functional polymorphisms for successful ageing, and by **Marco Malavolta (INRCA, Ancona, Italy**) who discussed the role of genetic factors in determining zinc dyshomeostasis in ageing with an emphasis on a flow cytometry-based assay to detect the NO-induced release of zinc from MT.

Since the elderly population is rapidly increasing, and the prevalence of overweight and obesity in elderly populations ranges widely upon geographical location and study design, **George Dedoussis **(**Harokopio University, Athens, Greece**) [3] reported the health status, blood and anthropometrical indices from 249 old Greek subjects (including nonagenarian) in relation to plasma zinc and dietary habits. Blood analysis confirmed the good health status of the elderly population recruited except for the high prevalence of obesity and hypercholesterolemia, which are in accordance with similar studies conducted in the elderly Greek populations [4].

A multi-disciplinary approach was used in the Zincage project in order to evaluate the relationship between zinc, psychosocial conditions and life-style in old healthy subjects. **Fiorella Marcellini **(**INRCA, Ancona, Italy**) [5] used a series of tests and questionnaires chosen with special regard to elderly people, including a life-style Questionnaire and a Food Frequency Questionnaire designed for the needs of Zincage in collaboration with **George Dedoussis**, the Mini Mental State Examination (MMSE), the Geriatric Depression Scale (GDS 15 items) and the Perceive Stress Scale (PSS). The results were an important outcome to evaluate the psychological differences among European elderly subjects (including nonagenarians) in relation with their zinc status. All psychological variables in the whole sample were related to physical zinc measurement. Among European countries, Greek elderly people, who were characterized by the lowest levels of plasma zinc, displayed also a significantly higher score both for PSS and GDS and a more marked age-dependent decline of MMSE when compared to other countries. In contrast, the old French population, which was characterized by the highest levels of plasma zinc, also had the lowest GDS and PSS scores and the highest score of MMSE compared with the other countries. A specific insight on lifestyle and psychological aspects of the Italian elderly, that confirmed the general trend shown in the whole sample, was further delineated in the "young scientists session" by **Cinzia Giuli **and **Roberta Papa **(**INRCA, Ancona, Italy**). Even if these results suggested that the zinc status is somehow positively associated with psychological variables, the rationale for promoting zinc supplementation in the elderly must be considered in the context of the possible neurotoxic effects of Zn^2+^[6]. Addressing the injurious role of zinc in neurological disease, **Stefano Sensi **(**University G. D'Annunzio, Chieti, Italy**) showed how excessive influx and/or mobilization of free zinc ions in neurons can promote the neuronal injury observed in cerebral ischemia, epilepsy, and brain trauma. The mechanisms by which zinc exerts its neurotoxicity include mitochondrial and extra-mitochondrial production of reactive oxygen species and disruption of metabolic enzyme activity, ultimately leading to activation of apoptotic and/or necrotic processes. Interestingly, in addition to acute neuronal injury, disturbances of zinc metabolism has also been demonstrated to play a role in the pathogenesis of Alzheimer's disease (AD) as the zinc cation acts as a potent trigger for beta-amyloid aggregation and plaque formation. Zinc is indeed found in high concentrations in mature amyloid plaques and its chelation has been found to effectively re-solubilize beta-amyloid from human postmortem AD plaques. These data suggest that the use of zinc supplements in elderly people should be considered only after a careful evaluation of the zinc status of the subjects as well as their genetic background particularly those genes related to the expression and mutation of proteins involved in Zn^2+ ^homeostasis.

### Session 2) Zinc and oxidative stress

In view of the fact that the release of zinc from MT represents an intracellular response to stress, biochemical modification of stress-related proteins might represent a useful target to influence zinc homeostasis and related mechanisms in ageing. **Alexander Bürkle **(**University of Konstanz, Germany) **[7,8] established a FACS-based immunoassay for the quantitative assessment for poly(ADP-ribose) polymerase 1, an abundant nuclear enzyme that binds via its zinc finger motifs to DNA with single or double strand breaks. The maximal level of PARP-1 activity is strongly correlated with life span of mammalian species, whereas within a given species maximal PARP-1 activity tends to decrease with donor age. Using this FACS-based immunoassay this group is addressing the possible link between PARP-1 activity and zinc status in humans. This aspect was extended by **Andrea Kunzmann **(**University of Konstanz, Germany**) who showed how cellular poly(ADP-ribosyl)ation capacity is modulated by *in vitro *zinc supplementation in PBMC.

As cellular poly(ADP-ribosyl)ation capacity is related to DNA damage and repair, it is relevant to study altogether these aspects in order to understand the impact on the functional status of the DNA repair mechanisms. With this aim, in the "young scientists session", **María Moreno-Villanueva **(**University of Konstanz, Germany**) addressed the influence of in *vitro *zinc on the repair capacity of Jurkat cells and human PBMC using an automated version of the fluorescence-detected alkali DNA unwinding (FADU) assay for the analysis of cellular DNA repair capacity. It appears that DNA repair capacity of PBMCs even from young, healthy donors can be increased by the simple addition of zinc in the physiological range. The results were very similar to the increase in poly(ADP-ribosyl)ation capacity.

A common cause of DNA damage and triggering of repair is the production of reactive oxygen species (ROS). **Jolanta Jajte **(**Medical University of Lodz, Poland**) [9] is assessing, by FACS and spectrofluorimetric method analysis, the modification of various cell components by ROS which are implicated in ageing and in numerous human diseases, such as cardiovascular disease, neurodegenerative processes and cancer. She analyzed the generation of ROS species in lymphocyte of healthy elderly subjects and atherosclerotic old patients and found a significant correlation between ROS generation and pathological changes in old atherosclerotic patients.

Protection against enhanced exposure to ROS, as it occurs in ageing, is mainly achieved by the gene expression of stress-related proteins such as Clusterin/Apoliprotein J (ApoJ). **Efstathios S. Gonos **(**National Hellenic Research Foundation, Athens, Greece**) approached ageing and longevity at the molecular level by studying the functions and the relevance for successful ageing of ApoJ. This has been recently identified as a novel survival factor [10] based on its cytoprotective function, which was demonstrated also by the sensitization of cell towards cytotoxicity resulting from suppression of ApoJ expression.

In the "young scientists session" **Ioannis P. Trougakos **(**National Hellenic Research Foundation, Athens, Greece**) further showed that ApoJ expression can be modulated by zinc and that ApoJ levels in the plasma may be used as a biochemical marker of zinc status.

Besides work on Apo J, the **Gonos **group set up an overall molecular and biochemical approach to understand proteasome functions in replicative senescence and cell survival, thus creating the basis for a possible investigation on the role of zinc in proteasome functions.

The accumulation of oxidatively modified proteins may represent an important hallmark of cellular ageing and pathology that is associated with oxidative/nitrosative stress. **Marco Colasanti **and **Giovanni Musci **(**University of Rome 3, Rome, Italy**) [11] determined the age-related changes occurring in MT and other zinc-dependent proteins, such as Specific Protein-1 (SP-1), showing for the first time that these proteins may be glutathionylated and that their important role in the adaptive cellular response to oxidative and nitrosative insults during ageing might thereby be lost. In addition, this group found that MT may also be differently glutathionylated upon ageing suggesting the presence of specific changes involving different MT isoforms.

Given the possibility that MT may become dysfunctional during ageing, corresponding changes in the activity of other zinc dependent enzymes might be found. Methionine sulfoxide reductase B (MsrB) is a zinc dependent protein-repairing enzymes that together with MsrA has the specific function of removing oxidatively-damaged methionine residues. **Bertrand Friguet **(**University Denis-Diderot, Paris, France) **[12,13] has developed enzymatic and immunochemical assay using cellular extracts from cells isolated from young and middle-aged individuals to monitor proteasome and methionine sulfoxide reductase activity and/or expression. The goal is to establish, within the ZincAge project, whether there is evidence for a correlation of the monitored parameters with age, gender and other factors relevant to zinc and oxidative stress.

Preliminary results, shown by **Filipe Cabreiro **(**University Denis-Diderot, Paris, France**), during the "young scientists session", on the effect of zinc on MOLT-4 cells overexpressing MsrA or MsrB demonstrate that increasing zinc concentration up to 50–75 μM lead to an increase in cells viability. Surprisingly, the addition of zinc seems also to decrease MsrA and MsrB activity, whereas chelating reagents have no effect on the metal content and on the activity of MsrB, thus suggesting a tight binding of the metal on the active site of this protein.

Molecular chaperones or stress proteins maintain the correct conformational homeostasis of proteins, protecting them against a number of stresses [14]. Proper function of these stress proteins is required for longevity [15]. Indeed, a hallmark of ageing is represented by an increase of aggregated and oxidized proteins. However, little is known about the causal relationship between protein misfolding, oxidative stress and chaperone induction. Moreover, the role of zinc as an immunomodulator and as an inducer of the stress response is not clear in this process. **Csaba Sőti **(**Semmelweis University, Budapest, Hungary**) [14] used the T-lymphocytic Jurkart cell line to analyze the relationship between protein misfolding, aggregation and chaperone induction. He found that, in contrast to the already known non-specific induction of heat shock proteins (Hsp70, Hsp90) caused by heat shock, oxidative stress increased expression of only certain Hsp proteins (Hsp90). Moreover, mild oxidative stress did not induce bulk protein denaturation and stress responses raising the question of how oxidative modification is able to induce chaperone expression. The mobilization of free zinc ions in response to oxidative stress might be involved in this phenomenon. Regarding the role of *in vitro *zinc on the modulation of chaperone expression in Jurkat cells, **Soti **showed that 12 μM was the optimal doses to induce chaperone expression and that higher zinc concentrations counteracted the heath shock and oxidative stress mediated induction of chaperones. Therefore, inhibition of chaperone expression might be one of the mechanisms involved in the toxicity of excessive amount of zinc.

Together with intracellular antioxidant and stress response proteins, an important role during ageing is assigned to the proteins involved in the extracellular antioxidant response. **Patrizia Mecocci **(**University of Perugia, Italy**) [16] measured the activities of a large spectrum of enzymatic antioxidants, such as plasma glutathione peroxidase (GPx), plasma catalase (Cat), plasma and erythrocyte superoxide dismutase (pSOD and eSOD) in subjects enrolled from different European geographical areas, including old subjects, atherosclerotic patients, nonagenarians, patients with infection and patients with cancer. Higher activity of GPx and pSOD was found in old subjects with atherosclerosis compared to old subjects with infection and to nonagenarians. The activity of GPx, Cat and pSOD did not differed with respect to gender, whereas eSOD was present higher values in women. The same enzyme displayed an age-associated increased activity, but the addition of zinc to erythrocytes from old donors *in vitro *was able to influence eSOD activity only slightly. Therefore, factors other than zinc, could be involved in the age-related up-regulation of eSOD activity. One of these factors may be the trace element copper which, in addition to zinc, is necessary for the catalytic activity of the enzyme. Therefore, further studies will be also undertaken to clarify the influence of the zinc/copper ratio on eSOD activity in elderly and nonagenarian subjects.

### Session 3) Zinc and genome stability

Dysfunctional telomeres trigger a stress response and provide molecular clues to the mechanisms linking growth suppression, zinc homeostasis and dysfunctional telomeres in mammalian cells.

**Maria Blasco **(**CNIO, Madrid, Spain**) [17], studying the effect of telomerase expression and telomere length on stem cell behaviour, demonstrated that critical telomere shortening has a negative impact on the proliferative capacity of bone marrow and neural stem cell and that telomere shortening in the absence of telomerase negatively impacts on the mobilization of epidermal stem cells. Using a microarray approach, **Blasco **showed also that overexpression of the catalytic subunit of telomerase (mTert), in primary murine cells, abrogates expression of the growth-inhibiting genes of the TGF-beta pathway and down-regulates some zinc-dependent transcription factors, such as Egr2, Klf4 and Zfp36. In contrast, the gene Txnip, involved in the redox-recycling of MT sulphur ligands, was found to be up-regulated by mTert overexpression. These results suggest that MT might also be involved in the stress response mediated by dysfunctional telomeres.

Gene array technology was also applied by **Dawn Mazzatti **and **Jonathan Powell **(**Unilever**, **Colworth, United Kingdom**) [18] to investigate the regulation of gene expression by zinc *in vitro*. Preliminary data indicate that several zinc-dependent genes (mainly specific MT isoforms and zinc transporters) and functional pathways are conserved between models, indicating their importance in zinc-mediated regulation of gene expression. The translation of these data to *in vivo *zinc supplementation is currently in progress.

Genome stability is controlled by a complicated network which involves many regulators. Zinc has been generally reported as a positive regulator of genome stability because it contributes to preventing DNA damage via modulation of the zinc dependent stress-response genes.

**Jolanta Jajte **and **Janusz Blasiak **(**Medical University of Lodz, Poland**) [9] found a significant correlation between DNA damage and DNA repair activity in lymphocytes of healthy elderly subjects and ill old atherosclerotic patients. Considering that increased levels of DNA damage is associated with the ageing process and that zinc status decreases with age, the capability of *in vitro *zinc to protect human PBMC from oxidative stress and DNA damage was tested. In contrast to the general protective effects shown in PBMC taken from young subjects, zinc was found to differently affect DNA damage induced by hydrogen peroxide in PBMC taken from old subjects. Thus, not all elderly subjects may respond in the same way to zinc supplementation perhaps due to a different genetic background. Therefore, genetic screening targeted to pro-inflammatory cytokines and MT genes might be the key to understanding the different behaviour of cells taken from elderly and identify old subjects who really need to be supplemented with zinc.

### Session 4) Zinc and signal triggering

Zinc plays an important role in cellular signalling and the zinc finger motifs which characterize several transcription factors are extraordinarily conserved among eukaryotic cells.

**Tamas Fulop **(**University of Sherbrooke, Canada**) [19] reviewed the multiple functions of zinc in biological systems, with an emphasis on the modulation of cytokine signal transduction in immune cells. Zinc can also influence other transcription factors by affecting DNA binding domain as well as their activation. Furthermore, zinc itself can also activate signalling molecules like calcium, by stimulating various pathways. Altogether these functions can modulate T cells response, proliferation and survival in response to pro-apoptotic signals. Thus, zinc deficiency observed with ageing can play a significant role in T cell activation changes and consequently in immunosenescence.

Since it is very well known that immune responses decline with ageing, **Audrey Varin **and **Georges Herbein **(**University of Franche-Comté, Besancon, France**) [20] studied the signal transduction, focusing on the activation of STAT pathway in T cells of elderly healthy subjects on exposure to cytokines (IL-2 and IL-6) and zinc stimulation. They found that with ageing there is an alteration in the Jak/STAT activation in response to IL-2 and IL-6 activation. Zinc supplementation *in vitro *slightly improved the age-related decrease depending on the age groups tested and the basal level of the Jak/STAT pathway activation.

**Daniela Monti **and **Rita Ostan **(**University of Florence, Italy**) [21] analyzed the role of zinc in cell proliferation and apoptosis affecting the development and integrity of the immune system. They studied the impact of the substitution (Arg to Pro) in the p53 codon, which affects apoptosis and cell cycle in an age-dependent manner. They also investigated the relationship between apoptosis and zinc treatment. They found that in arginine/arginine carriers zinc is able to decrease early apoptosis and to increase late apoptosis/necrosis. In contrast, no effect was detected in proline/proline or proline/arginine carriers. Furthermore, zinc treatment induces decreased S phase commitment and concomitant increase of the cells in the G2/M phase in old subjects treated with different concentrations of zinc. These results suggest that functional polymorphisms other than MT and cytokine genes may be involved in the individual response to zinc supplementation. Thus, future efforts will also be directed towards the evaluation of apoptosis and cell-cycle control in old supplemented individuals in relationship to p53 polymorphisms.

### Session 5) Zinc and immune mediators

Long term clonal cultures represent good models for studying the behaviour of T cells under chronic antigenic stress and facilitate the in vitro testing of interventions in a longitudinal ageing system. **Graham Pawelec **(**University of Tübingen, Germany**) [22] described the effect of zinc supplementation on growth characteristics surface molecule expression, cytokine production and heat shock protein expression of human CD4+ T cell clones (TCC) derived from young and old donors, including centenarians. The results suggested that at least for T cells, the impact of zinc over-supplementation may be limited to decreased cell growth rates, perhaps due to increased apoptosis, but that surviving cells are unlikely to be functionally compromised. Of particular interest, **Sven Koch **(**University of Tübingen, Germany**) [23] from the same **Pawelec **group has investigated the CMV repertorie in the elderly using MHC/peptide multimers in different European populations, including Zincage donors (62–90 yrs. old). He found that T cells specific for CMV specific, such as CD8+CD45RA^-^CCR7^-^CD28^-^CD57+ terminally differentiated phenotype cells, express high levels of the inhibitory killer lectin-like receptor KLRG1. However, high KLRG1 expression and CMV IgG titre did not correlate significantly with increasing ageing suggesting that KLRG1 may be a valuable biomarker of immunosenescence. Therefore, the deletion of CMV-specific KLRG1/CD57-double positive dysfunctional CMV-specific cells might be of benefit in at-risk elderly. The impact of zinc in this CD8+ cell populations will be assessed in old zinc supplemented individuals.

The role of the thymus is vital for orchestration of T cell development and maturation.**Richard Aspinal and Wayne Mitchell (Imperial College London, UK) **[24] evaluating the role played by zinc in maintaining thymic output in healthy elderly individuals used FACS analysis and real time PCR technology. They carried out experiments on thymic output in healthy individuals aged between 60–90 years, prior to supplementation with zinc. They analyzed 300 individuals for the total T cell numbers (CD3) and recent thymic emigrants (TRECs). The results indicate that TREC levels are stable between countries but females have slightly higher values compared to male. Further efforts will be addressed to understand the effects of zinc supplementation on thymic output in elderly.

Cellular components of both the adaptive and innate immune system produce different cytokines and chemokines which modulate effector function during the immune response. Accordingly, **Erminia Mariani **(**Istituti Ortopedici Rizzoli, Bologna, Italy**) [25,26] evaluated the concentration of three CC type chemokines (MCP-1, MIP-1alpha and RANTES), a CXC type chemokine (IL-8) and two pro-inflammatory cytokines (IL-6 and TNF-alpha) in human plasma samples obtained from subjects of various age and from the different European countries. The circulating levels of each soluble factor, analysed in pooled samples from old healthy subjects were significantly correlated with each other and with plasma zinc, suggesting that being high or low producers, at least for these factors, is a distinct intrinsic feature of each individual and may be related to the individual regulation of zinc homeostasis. There was also a general trend to increases in all these soluble factors under pathological conditions, including obesity, suggesting an inflammatory status in pathology independently of the age of the subjects.

Since experimental human zinc deficiency is known to decrease Th1 but not Th2 immune response, **Lothar Rink **(**Aachen University, Germany**) [27,28] investigated the effect of a preliminary supplementation trial with zinc in Germany on the Th1/Th2 balance and on T cell activation. Preliminary results showed that the number of CCR4 (TH2) and CCR5 (TH1)-positive T cells did not change significantly after zinc treatment, whereas the number of activated T cells (CD4+/CD25+) was significantly decreased. Additionally, a method to quantify zinc importer (hZIP), zinc exporter (ZnT) and intracellular free zinc by flow cytometry was established. The altered expression profile of these transporters and their homeostasis has a direct influence on the cells. Down regulation of hZIP seems to be a prerequisite for immortalisation, whereas up-regulation of hZIP favoured apoptosis in T cell. Despite the possibility that the effect of zinc in cells from elderly subjects might sometimes be to induce and sometimes to prevent apoptosis or DNA damage, depending on the doses and individual characteristics, there is a general agreement that zinc supplementation with physiological doses is generally beneficial for the individuals. One explanation for this potential paradox might be found in the hormetic theory. Hormesis in ageing, as discussed in details by **Suresh Rattan **(**University of Aarhus, Denmark**) [29], is characterized by the beneficial effect that results from the cellular response to mild stress. His studies have shown that repeated mild heat stress (RMHS) has anti-ageing effect on growth and various other cellular and biochemical characteristics of normal human skin fibroblasts undergoing ageing in vitro. RMHS has also been tested in combination with potential hormetic molecules, such as curcumin, on ageing and longevity of human cells in culture.

In contrast to mild stress, the life long antigenic burden leads to a condition of chronic inflammation with increased lymphocyte activation and pro-inflammatory cytokine production. In this context, **Calogero Caruso **(**University of Palermo, Italy**) [22,30] has studied zinc availability in old subjects with coronary artery disease, healthy controls and centenarians with respect to a pro-inflammatory or anti-inflammatory genotype. In age-related diseases zinc seems to influence, through bio-molecular pathways, inflammatory mediators that, in turn, influence zinc availability. The results confirmed that the zinc sequestering proteins, such as MT, at least in ageing show a correlation with inflammation.

This last aspect may be also relevant in non-pathological conditions, taking into account that chronic antigenic load is the major driving force of immunosenescence

Finally, **Rafael Solana **(**University of Cordoba, Spain) **[22,31] showed that the T cell subpopulation CD8+CD28null, characterized by high NK associated receptor expression and effector memory (EM) phenotype, is expanded in elderly donors when compared to young and that this expansion is associated with CMV seropositivity and to increased risk of death. It has been described that CMV is major inducer of oligoclonal expansion in ageing. **Solana **found a significant expansion both in young controls and healthy elderly donors after antigenic stimulation. This introduces the idea that the expansion of a subset (EM2) of CD8+ T cells in the elderly is due to a biased differentiation after chronic stimulation of the immune system somehow driven by persistent antigens such CMV. The possibility that the individual's zinc status might help to keep under the undesired effects of persistent viruses like CMV under control, will also be investigated during the ZincAge Project.

## Conclusion

Improving our knowledge of the many factors related to the control of zinc homeostasis and immune efficiency in ageing is necessary in order to develop frailty prevention programmes based on zinc supplementation. In fact, it is clear that zinc is of extraordinary and diverse importance in human biology and nutrition but, especially in ageing, zinc research is still in a relatively immature stage of development. New knowledge on the function of metallothioneins, zinc transporters and on the effect of the genetic background has been acquired or is emerging, based on innovative methods and technologies for the assay of zinc ion availability and the related activity of zinc-dependent enzymes. The answer to many scientific questions regarding the characterization of the benefits/risks of zinc supplementation in the elderly and of zinc responsive biological systems, including oxidative stress and host defence against infection, will hopefully be attained after extensive discussion of the results at the end of the Zincage Project, with a particular focus on the different dietary habits in various European countries. Indeed, different diets associated with zinc status may be recommended at the end of the Project in order to maintain zinc homeostasis and consequently to maintain good health status and achieve longevity.

## References

[B1] http://www.zincage.org.

[B2] Mocchegiani E, Costarelli L, Giacconi R, Cipriano C, Muti E, Tesei S, Malavolta M (2006). Nutrient-gene interaction in ageing and successful ageing A single nutrient (zinc) and some target genes related to inflammatory/immune response. Mech Ageing Dev.

[B3] Kaliora AC, Dedoussis GV, Schmidt H (2006). Dietary antioxidants in preventing atherogenesis. Atherosclerosis.

[B4] Trichopoulou A, Gnardellis C, Lagiou A, Benetou V, Trichopoulos D (2000). Body mass index in relation to energy intake and expenditure among adults in Greece. Epidemiology.

[B5] Marcellini F, Giuli C, Papa R, Malavolta M, Mocchegiani E (2006). Psychosocial Aspects and Zinc Status: Is There a Relationship with Successful Ageing?. Rejuvenation Research.

[B6] Sensi SL, Jeng JM (2004). Rethinking the excitotoxic ionic milieu: the emerging role of Zn(2+) in ischemic neuronal injury. Curr Mol Med.

[B7] Bürkle A, Diefenbach J, Brabeck C, Beneke S (2005). Ageing and PARP. Pharmacol Res.

[B8] Bürkle A, Brabeck C, Diefenbach J, Beneke S (2005). The emerging role of poly(ADP-ribose) polymerase-1 in longevity. Int J Biochem Cell Biol.

[B9] Jajte JM (1997). Chemical-induced changes in intracellular redox state and in apoptosis. Int J Occup Med Environ Health.

[B10] Trougakos IP, Lourda M, Agiostratidou G, Kletsas D, Gonos ES (2005). Differential effects of clusterin/apolipoprotein J on cellular growth and survival. Free Radic Biol Med.

[B11] Musci G, Persichini T, Casadei M, Mazzone V, Venturini G, Ponticelli F, Colasanti M (2006). Nitrosative/oxidative modifications and ageing. Mech Ageing Dev.

[B12] Cabreiro F, Picot CR, Friguet B, Petropoulos I (2006). Methionine sulfoxide reductases: relevance to aging and protection against oxidative stress. Ann N Y Acad Sci.

[B13] Petropoulos I, Friguet B (2005). Protein maintenance in aging and replicative senescence: a role for the peptide methionine sulfoxide reductases. Biochim Biophys Acta/Proteins and Proteomics.

[B14] Sőti C, Csermely P (2003). Ageing and molecular chaperones. Exp Gerontol.

[B15] Sőti C, Pál C, Papp B, Csermely P (2005). Molecular chaperones as regulatory elements of cellular networks. Curr Opin Cell Biol.

[B16] Mecocci P, Polidori MC, Troiano L, Cherubini A, Cecchetti R, Pini G, Straatman M, Monti D, Stahl W, Sies H, Franceschi C, Senin U (2000). Plasma antioxidants and longevity: a study on healthy centenarians. Free Radic Biol Med.

[B17] Geserick C, Tejera A, Gonzalez-Suarez E, Klatt P, Blasco MA Expression of mTert in primary murine cells links the growth-promoting effects of telomerase to transforming growth factor-beta signaling. Oncogene.

[B18] Hu HL, Forsey RJ, Blades TJ, Barratt ME, Parmar P, Powell JR (2000). Antioxidants may contribute in the fight against ageing: an in vitro model. Mech Ageing Dev.

[B19] Larbi A, Douziech N, Dupuis G, Khalil A, Pelletier H, Guerard KP, Fulop T (2004). Age associated alterations in the recruitment of signal-transduction proteins to lipid rafts in human T lymphocytes. J Leucoc Biol.

[B20] Fulop T, Larbi A, Douziech N, Levesque I, Varin A, Herbein G (2006). Cytokine receptor signalling and aging. Mech Ageing Dev.

[B21] Alberti S, Cevenini E, Ostan R, Capri M, Salvioli S, Bucci L, Ginaldi L, De Martinis M, Franceschi C, Monti D (2006). Age-dependent modifications of Type 1 and Type 2 cytokines within virgin and memory CD4(+) T cells in humans. Mech Ageing Dev.

[B22] Pawelec G, Akbar A, Caruso C, Solana R, Grubeck-Loebenstein B, Wikby A (2005). Human immunosenescence: is it infectious?. Immunol Rev.

[B23] Koch S, Solana R, Rosa OD, Pawelec G (2006). Human cytomegalovirus infection and T cell immunosenescence. Mech Ageing Dev.

[B24] Aspinall R (2006). T cell development, ageing and Interleukin-7. Mech Ageing Dev.

[B25] Mariani E, Meneghetti A, Neri S, Ravaglia G, Forti P, Cattini L, Facchini A (2002). Chemokine production by natural killer cells from nonagenarians. Eur J Immunol.

[B26] Pulsatelli L, Meliconi R, Mazzetti I, Dolzani P, Meneghetti A, Neri S, Silvestri T, Ravaglia G, Forti P, Facchini A, Mariani E (2000). Chemokine production by peripheral blood mononuclear cells in elderly subjects. Mech Ageing Dev.

[B27] Cakman I, Rohwer J, Schütz R-M, Kirchner H, Rink L (1996). Dysregulation between TH1 and TH2 T cell subpopulations in elderly persons. Mech Ageing Dev.

[B28] Ibs KH, Rink L (2003). Zinc-altered immune function. J Nutr.

[B29] Rattan SIS (2004). Aging intervention, prevention and therapy through hormesis. J Gerontol Biol Sci.

[B30] Caruso C, Lio D, Cavallone L, Franceschi C (2004). Aging, longevity, inflammation, and cancer. Ann N Y Acad Sci.

[B31] Solana R, Casado JG, Delgado E, Delarosa O, Marin J, Duran E, Pawelec G, Tarazona R Lymphocyte activation in response to melanoma: interaction of NK-associated receptors and their ligands. Cancer Immunol Immunother.

